# Supplementary material for: Early onset of neurological features differentiates two outbreaks of Lassa fever in Ebonyi state, Nigeria during 2017–2018
Source: PLoS Negl Trop Dis. 2021 Mar 8;15(3):e0009169. doi: 10.1371/journal.pntd.0009169 (PMC7984835; doi:10.1371/journal.pntd.0009169)
Supplement: S1 Table — (DOCX) [file pntd.0009169.s001.docx]

| **S1 Table. Clinical Stages of Severe Lassa Fever (Adapted from WHO 2016) [** WHO. Clincial Management of Patients with Viral Haemorrhagic Fever: A Pocket Guide for the Front-line Health Worker. World Heal Organ. 2016;1–191**]^1^** | |
| --- | --- |
| **Stage** | **Symptoms** |
| 1 (Days 1-3) | • General weakness and malaise Day  • High Fever, >39^0^C constant with peaks of 40-41^0^C |
| 2 (Days 4-7) | • Sore throat with (white exudative patches) very common.  • Headache; back, chest, side, or abdominal pain.  • Conjunctivitis  • Nausea and vomiting  • Diarrhea  • Proteinuria  • Productive cough  • Low blood pressure (systolic <100mmHg)  • Anemia |
| 3 (After Day 7) | • Oedema of the face and neck  • Convulsions  • Mucosal bleeding (mouth, nose, eyes)  • Internal bleeding  • Encephalopathy with confusion or disorientation |
| 4 (After Day 14) | • Coma  • Death |
