# Supplementary material for: Early onset of neurological features differentiates two outbreaks of Lassa fever in Ebonyi state, Nigeria during 2017–2018
Source: PLoS Negl Trop Dis. 2021 Mar 8;15(3):e0009169. doi: 10.1371/journal.pntd.0009169 (PMC7984835; doi:10.1371/journal.pntd.0009169)
Supplement: S2 Table — (DOCX) [file pntd.0009169.s002.docx]

**S2 Table: NCDC Case Definitions [**Nigeria Centre for Disease Control (NCDC).

Standard Operating Procedures for Lassa Fever Case Management. 2017]^2^

| **Alert case**  Any person who has an unexplained fever  (i.e. Malaria and other likely causes of fever have been ruled out), with or without bleeding  OR  Any person who died after an unexplained severe illness with fever and bleeding |
| --- |
| **Suspected case**  An illness of gradual onset with one or more of the following: malaise, fever, headache, sore throat, cough, nausea, vomiting, diarrhea, myalgia (muscle pain), central chest pain or retrosternal pain, hearing loss and either :  a. History of contact with excreta or urine of rodents  OR  b. History of contact with a probable or confirmed Lassa fever case within a period of 21 days of onset of symptoms  OR  Any person with inexplicable bleeding/ hemorrhaging |
| **Probable case**  Any suspected case as defined above but who died without collection of specimen for laboratory testing |
| **Confirmed case** Any suspected case with laboratory confirmation (positive IgM antibody, PCR or virus isolation). |
| **Alert threshold**  A single suspected case of Lassa fever. The outbreak threshold is a single confirmed case of Lassa fever |
