# Supplementary material for: Early onset of neurological features differentiates two outbreaks of Lassa fever in Ebonyi state, Nigeria during 2017–2018
Source: PLoS Negl Trop Dis. 2021 Mar 8;15(3):e0009169. doi: 10.1371/journal.pntd.0009169 (PMC7984835; doi:10.1371/journal.pntd.0009169)
Supplement: S3 Table — (DOCX) [file pntd.0009169.s003.docx]

**S3 Table: Ribavirin Treatment Regimen (McCormick Regimen)**

| **Ribavirin Regimen (McCormick Regimen)** | | |
| --- | --- | --- |
| Period | Dose | Frequency |
| Loading Dose | 33mg/kg (maximum dose of 2.64 g) | Stat |
| Day 1-4 | 16mg/kg (maximum dose of 1.28 g) | 6 hourly |
| Day 5-10 | 8mg/kg (maximum dose of 0.64g) | 8 hourly |
