# Supplementary material for: Early onset of neurological features differentiates two outbreaks of Lassa fever in Ebonyi state, Nigeria during 2017–2018
Source: PLoS Negl Trop Dis. 2021 Mar 8;15(3):e0009169. doi: 10.1371/journal.pntd.0009169 (PMC7984835; doi:10.1371/journal.pntd.0009169)
Supplement: S4 Table — (DOCX) [file pntd.0009169.s004.docx]

**S4 Table : Clinical symptoms significantly associated with mortality in the two outbreaks**

| Characteristics | | Survived | | | Died | | | Relative Risk (95%CI)* | P value |  |  |  |
| --- | --- | --- | --- | --- | --- | --- | --- | --- | --- | --- | --- | --- |
|  | | N | | % | | n | % |  |  |  |  |  |
| Total | | 49 | | 60.5 | | 32 | 39.5 |  |  |  |  |  |
| Outbreak 1  Dec 2017 – Apr, 2018 | | 47 | | 70.1 | | 20 | 29.9 | Reference | <0.001** |  |  |  |
| Outbreak 2  Aug 2018 – Dec, 2018 | | 2 | | 14.3 | | 12 | 85.7 | 4.9 (1.3-17.8) |  |  |  |  |
| **Presenting symptoms**  **General** | | | | | | | | | |  | |  |
| Any Neuro symptom | No | 35 | | 92.1 | | 3 | 7.9 | Reference | <0.001** |  |  |  |
|  | Yes | 14 | | 32.6 | | 29 | 67.4 | 8.5 (2.8-24.8) |  |  |  |  |
| Fever | No | 7 | | 100.0 | | 0 | 0.0 | Reference | 0.025** |  |  |  |
|  | Yes | 42 | | 56.8 | | 32 | 43.2 | 1.7 (1.4-2.1) |  |  |  |  |
| Headache | No | 14 | | 82.4 | | 3 | 17.6 | Reference | 0.046** |  |  |  |
|  | Yes | 34 | | 55.7 | | 27 | 44.3 | 1.4 (1.0-2.0) |  |  |  |  |
| Red eye | No | 36 | | 75.0 | | 12 | 25.0 | Reference | 0.003** |  |  |  |
|  | Yes | 13 | | 41.9 | | 18 | 58.1 | 1.7 (1.1-2.7) |  |  |  |  |
|  | Yes | 14 | | 53.8 | | 12 | 46.2 | 1.2 (0.8-1.8) |  |  |  |  |
| Oedema | No | 39 | | 86.7 | | 6 | 13.3 | Reference | 0.001** |  |  |  |
|  | Yes | 10 | | 34.5 | | 19 | 65.5 | 2.5 (1.5-4.2) |  |  |  |  |
| Bleeding | No | 30 | | 81.1 | | 7 | 18.9 | Reference | 0.001** |  |  |  |
|  | Yes | 19 | | 43.2 | | 25 | 56.8 | 1.8 (1.2-2.7) |  |  |  |  |
| Respiratory distress | No | 15 | | 100.0 | | 0 | 0.0 | Reference | 0.001** |  |  |  |
|  | Yes | 34 | | 55.7 | | 27 | 44.3 | 1.7 (1.4-2.2) |  |  |  |  |
| **Neurological** | | | | | | | | | |  | | |
| Neck pain | No | 42 | | 68.9 | | 19 | 31.1 | Reference | 0.007** |  |  |  |
|  | Yes | 6 | | 33.3 | | 12 | 66.7 | 2.0 (1.0-4.0) |  |  |  |  |
| Ear pain | No | 47 | | 67.1 | | 23 | 32.9 | Reference | 0.002** |  |  |  |
|  | Yes | 2 | | 18.2 | | 9 | 81.8 | 3.69 (1.0-13.0) |  |  |  |  |
| Seizure | No | 40 | | 80.0 | | 10 | 20.0 | Reference | <0.001** |  |  |  |
|  | Yes | 9 | | 29.0 | | 22 | 71.0 | 2.7 (1.5-4.8) |  |  |  |  |
| Altered consciousness | No | 43 | | 86.0 | | 7 | 14.0 | Reference | <0.001** |  |  |  |
|  | Yes | 6 | | 19.4 | | 25 | 80.6 | 4.4 (2.1-9.1) |  |  |  |  |
| **Laboratory Parameters** | | | | | | | | | |  | | |
| Creatinine (umol/l) | <53 | 4 | | 100.0 | | 0 | 0.0 |  |  |  |  |  |
|  | *53-106* | 29 | | 61.7 | | 18 | 38.3 |  | 0.194 |  |  |  |
|  | >106 | 16 | | 53.3 | | 14 | 46.7 |  |  |  |  |  |
| Urea (mm/L) | <2.5 | 2 | | 100.0 | | 0 | 0.0 |  |  |  |  |  |
|  | 2.5-7.9 | 41 | | 65.1 | | 22 | 34.9 |  | 0.067 |  |  |  |
|  | >7.9 | 6 | | 37.5 | | 10 | 62.5 |  |  |  |  |  |
| AST (mmol/l) | <11 | 1 | | 100.0 | | 0 | 0.0 |  |  |  |  |  |
|  | 11-38 | 21 | | 60.0 | | 14 | 40.0 |  | 0.718** |  |  |  |
|  | >38 | 27 | | 60.0 | | 18 | 40.0 |  |  |  |  |  |
| ALT (mmol/l) | <10 | 6 | | 100 | | 0 | 0.0 |  |  |  |  |  |
|  | 10-47 | 36 | | 64.3 | | 20 | 35.7 |  | 0.013** |  |  |  |
|  | >47 | 7 | | 36.8 | | 12 | 63.1 |  |  |  |  |  |
| Platelet count | <150 | 17 | | 51.1 | | 16 | 48.5 |  | 0.171** |  |  |  |
|  | 150-450 | 32 | | 66.7 | | 16 | 33.3 |  |  |  |  |  |
|  | Creatinine (umol/l)  [median(IQR)] | | 95(67-121) N=40 | | | 180(101-350) N=20 | |  | <0.001*** |  |  |  |
|  | AST (mmol/l) [median (IQR)] | | 52(30.5-8) N=41 | | | 89(49-89) N=20 | |  | 0.022*** |  |  |  |
|  | Urine output  (mL/m^2^/24hrs)  [mean(SD)] | | 1.3(1.2) N=49 | | | 3.8(3.4) N=32 | |  | 0.001* |  |  |  |
|  | Platelet count  (x10^9^/l)  [mean (SD)] | | 173.2(82.1) N=41 | | | 98.8(48.8) N=19 | |  | 0.001* |  |  |  |
| Days between onset of symptoms and admission | Median  (IQR) | | 7(3-10) N=49 | | | 10(6-14) N=19 | |  | <0.001*** |  |  |  |

*CI, confidence interval; IQR, interquartile range; AST: Aspartate aminotransaminase

* T-test, ** Chi squared test, *** Kruskal Wallis test.
