# Supplementary material for: Early onset of neurological features differentiates two outbreaks of Lassa fever in Ebonyi state, Nigeria during 2017–2018
Source: PLoS Negl Trop Dis. 2021 Mar 8;15(3):e0009169. doi: 10.1371/journal.pntd.0009169 (PMC7984835; doi:10.1371/journal.pntd.0009169)
Supplement: Attachment — Submitted filename: RESPONSE TO REVIEWERS.docx [file pntd.0009169.s005.docx]

RESPONSE TO REVIEWERS

The editor PLOS NTD, below are my responses to the reviewers comment on my Manuscript titled : **EARLY ONSET OFNEUROLOGICAL FEATURESDIFFERENTIATES TWO OUTBREAKSOF LASSA FEVER IN EBONYI STATE,NIGERIA DURING 2017-2018**

<b>Editorial and Data Presentation Modifications?</b></br><br/>

1. Reviewer #3: Line 73:  These are fairly old references that made a lot of assumptions of the asymptomatic burden within the population based on seroprevalence studies at the time.  I'm not sure they are entirely accurate and would recommend the authors mute or blunt this statement.

**The statement was blunted to read " LF symptoms may be mild or asymptomatic in about 80% of cases, but can otherwise cause acute illness"**

2. Line 84:  Hospitalized case CFR in Nigeria or Sierra Leone?  They are recognized to be different by those working in the field, likely due to differential standard of care in each setting.  Perhaps viral genotypes as well, but not enough data to confirm.

**"Case fatality rate of up to 20% of cases in hospitalized patients increasing as high as 50% during epidemics was deleted"**

3. Line 119:  AEFUTHA first use, spell out acronym.

**It has been fully spelt out " Alex Ekwueme Federal University Teaching Hospital Abakaliki (AEFUTHA)".**

4. Line 200:  Readers would benefit by understanding a better definition of what a "trader" does.  Is this a highly transient merchant that spends time in both rural and developed areas?

"**The traders consist of adults and youths in low- and middle-income social classes who engage in wholesale and retail buying/selling of beverages, food stuff, electronics, stationary and textile materials"**

5. Line263-264:  Given the high OR of farmers from either outbreak, readers would benefit from understanding if any differences in time to presentation between outbreaks (i.e. are farmers from rural settings showing  up later to the hospital than farmers from more urban areas?).  It is well established that persons from more rural settings are less likely to seek medical attention and likely present due to unfortunate issues associated with limited transportation access, social stigmas, or even fears of treatment (ie risks of going to hospital). 
Would also be wise to recognize the role of increased/improved surveillance or even public health outreach campaigns in either outbreak setting?  Are there differences in the public education programs in these two settings?

**"The median time between symptom onset and admission was 8 (IQR: 3-11) for traders and 7 (IQR: 6-14) for farmers. Farmers in outbreak 1 had a median time between onset of symptoms of presentation of 6.5 (IQR:5-14.5) whereas for farmers in outbreak 2 this was 12 (IQR:7-12) but data was missing for 2 patients. Obviously, farmers from the rural setting, showed up later to the hospital than those from the urban probably due to socio-cultural factors and transportation challenges. Also, there is improved public health campaign and surveillance in the urban area."**

6. Line 301:  It's possible that for the area of Ebonyi State, that the neurological presentation timelines associated with increased mortality are unusual; but by enlarge,these features are common to severe LF in humans.  I would recommend muting this statement or making it very specific to the geography of this study.

**"This study revealed an unusual form of presentation with a different profile of signs and symptoms and a high mortality rate in the rural setting of our locality".**

7. A map of the two outbreak sites would help the reader understand the geographic separation between the outbreaks and support the potential for different viral isolates to be be responsible for these two outbreaks.

**Map has been included**


<b>Summary and General Comments</b></br></br>


1.      Given the high genetic diversity of Lassa virus in Nigeria and with at least three known clades documented in this country it is important to provide some genetic analysis on these cases to minimally determine the infecting clade. The increases in neurological manifestations may be due to a differing clade of virus, which in itself would be highly interesting.

**"Unfortunately, due to poor storage of the samples, phylogenetic analysis could not be carried out for the cases in outbreak 2 as the intergrity of the samples has been compromised from power outage".**

2.      I suggest adding a map of Nigeria, highlighting Ebonyi state as well as other regions in Nigeria that were experiencing Lassa fever outbreaks during this time period.

**"Map has been included"**

3.      The definition of a confirmed and probable case must be included in the text.

**"Probable case is any suspected case as defined above but who died without collection of specimen for laboratory testing. Confirmed case is any suspected case with laboratory confirmation (positive IgM antibody, PCR or virus isolation)."**

4.      Please describe the molecular diagnostics utilized in identifying LF cases. One vs two target, what gene/region was amplified, etc.

**Methodology for LASV Sample handling**

**3-5 mls of blood sample was collected from each suspected Lassa patient in EDTA bottle and transported to the Virology laboratory in a cold chain in triple packaged condition in line with NCDC guideline. The plasma was harvested from each sample into a plain bottle in a glove box. The plasma samples were inactivated and RNA extracted through spin column method using QIAamp RNA Mini Kit (**[**www.qiagen.com**](http://www.qiagen.com)**) in strict compliance with the manufacturer’s instruction. Sample inactivation was done inside a glove box while the buffer washings and elution were done on the beach. Altona 2.0 Lassa diagnostic kits that targets the S-gene (GPC) and L-gene of Lassa virus was used for amplification using MIC qPCR cycler. Thermo-cycling conditions were set at 55 C for 20minutes (Reverse Transcription), 95 C for 2minutes (Denaturation) and 45 amplification cylces at 95 C for 15, 55 C for 45 sec and 72 C for 15sec sequentially. Cycle threshold (CT) value was set at 40. Detection of both genes was interpreted as Lassa PCR positive result if Negative test control (NTC) and Positive test control (PTC) are Negative and Positive respectively, and internal controls are in order.**

5.      Did other concurrent outbreaks of LF in Nigeria also observe increased neurological manifestations?

**"Some previous studies on LF outside this environment have demonstrated early neurological signs with positive outcomes, poor outcomes, as well as late neurological manifestations but not concurrently".**

6.      The benefit of ribavirin in treating LF cases is highly controversial, possibly due in part to a lack of publications outlining its use. Please discuss any benefits of ribavirin treatment observed in patients from this study.

**"Whereas early commencement of treatment influenced the outcome of the cases in outbreak 1, high fatality rate was observed in outbreak 2 even amongst asymptomatic care givers that later developed symptoms while in the hospital presenting with neurological symptoms right from onset . This underscores the import of the second outbreak."**

7.      The data tables and associated statistics should be re-done to differentiate probable and confirmed cases. Alternatively, you could exclude the probable cases from these tables since you did not confirm them as Lassa positive / LF cases. The statistics are misleading when everything is grouped together.

**The table for the probable and confirmed cases has been separated**

8.      Please define the abnormal laboratory tests. Other laboratory tests run should also be mentioned, even if they do not differ between the two outbreaks
**"Abnormal laboratory findings identified include elevated serum creatinine, AST, ALT and thrombocytopenia. These has been demonstrated as predictors of mortality in previous studies"**

Minor comments:
1.      Once defined after first use, please use LF and LASV throughout the manuscript

**Changes effected**

2.      Outbreak does not need to be capitalized

**Changes effected**

3.      What is a mini morgue?

**"A miniature mortuary outside the ward, where corpses are kept temporarily while arrangements are made for a safe burial".**

4.       Please specify the statistical tests used in the table footnote so that readers do not have to search for it.

**Changes effected**

Reviewer #2: Chika-Igwenyi and co-workers describe the epidemiology, socio-demographics, clinical characteristics, and outcomes of patients seen during two Lassa fever outbreaks in Ebonyi State, Nigeria between December 2017 and 39 December 2018. The results are important, but a few improvements in data presentation are needed.

5. Table 3 shows much of the same data as Table 2 can be eliminated or placed in Supplemental Material.

**Changes effected**

The normal range for clinical parameters, such as creatinine and AST, should be provided in Tables 2 and 3. Elevated creatinine and AST have been observed in other Lassa fever clinical cohorts in Nigeria and Sierra Leone. AST levels in the current study appear closer to the normal range. These prior studies and differences/similarities should be discussed.

**Abnormal laboratory findings identified include elevated serum creatinine, AST, ALT and thrombocytopenia. These has been demonstrated as predictors of mortality in previous studies**

Headers should be added to Table 4 to indicate the different analyses.

**Changes effected**

Reviewer #3: Dr. Chika-Igwenyi and colleagues provide an epidemiological analysis of two outbreaks in Ebonyi State, Nigeria from 2018-2019.  The work is strictly epidemiological with support of clinical information based on disease severity and associated symptoms of disease.  The central claim appears to be that between the two outbreaks, different disease manifestations surfaced that were associated with differential mortality outcomes.  Unfortunately there is no genetic information of the virus isolates associated with either outbreak, thus making interpretation of the possibility of two different viruses variants being responsible for the difference in outcome.  LASV infection classically is associated with late onset neurological signs of which chances for poor outcome are increased if no supportive therapy or pharmaceutical interventions are started.  The work might also be assisted by reporting CT values (or genomic equivalent values if possible) of the real-time
RT-PCR to get a better idea of what state the persons were in upon admission to the hospital for treatment.  As mentioned elsewhere in this review, it is not uncommon for persons in rural setting to delay seeking treatment for mainly socio-economic reasons, so it was not at all surprising to see more severe disease manifestations in that population.  But I think more data related to disease state and viral burden, if not the phylogenetic analysis would be necessary to confidently  ascertain these outbreaks were indeed caused by different.

**"Ebonyi State is one of the three high burdened states with frequent outbreaks of LF [39-41]. NCDC reported an epidemiological analysis and clinical aspects of the LF outbreak that occurred in Nigeria during January 1–May 6, 2018. Among the 3 states with the highest number of cases, the positive rates were 16.5% (Edo), 31.6% (Ondo), and 69.6% (Ebonyi). Ebonyi recorded the highest positive rate and among the 3 most affected states, CFR was 14.6% (Edo), 24.2% (Ondo), and 23.4% (Ebonyi)[41]".**

Also made the following changes

- Added notes on the test used to generate the p values at the bottom of the tables.
- Added a table on the differences between probable and confirmed.
- Lab results in terms of numbers within the normal range.
- Median time to presentation by occupation in the text.
- Titles for the table with the logistic regression.
- Included one of the authors mistakenly missed out during submission.

Chika-Igwenyi Nneka

Corresponding Author
